# Generation Characteristics and Regulation Mechanisms of Monodisperse Droplets of JP-10-Based Nanofluids via Drop-on-Demand Technology

**DOI:** 10.3390/nano16161001

**Published:** 2026-08-14

**Authors:** Bingzheng Wang, Tianhang Wang, Zixuan Zhou, Hui Wang, Shengji Li, Xuefeng Huang

**Affiliations:** 1 Institute of Energy, Department of Physics, Hangzhou Dianzi University, Hangzhou 310018, China; w15663061350@sina.com (B.W.); 19157700638@163.com (T.W.); zzx1035480524@gmail.com (Z.Z.); zqwhrs@163.com (H.W.); 2 College of Materials and Environmental Engineering, Hangzhou Dianzi University, Hangzhou 310018, China

**Keywords:** JP-10-based nanofluids, drop-on-demand, monodisperse droplets, droplet breakup mechanism, phase Doppler anemometry

## Abstract

JP-10 is a pivotal high-density hydrocarbon fuel for advanced aerospace propulsion systems. Doping aluminum nanoparticles to prepare nanofluid fuels is a promising route to enhance its energy density and combustion performance, yet the droplet formation mechanism of such multiphase fuels remains poorly understood, hindering single-droplet combustion research and atomization system optimization. This work constructed a piezoelectric drop-on-demand (DOD) monodisperse droplet generation platform integrated with phase Doppler anemometry (PDA) and high-speed imaging. Using Al/JP-10/OA nanofluids with aluminum mass fractions of 0.1 wt. %, 0.5 wt. % and 1.0 wt. %, we systematically explored the effects of liquid flow rate, driving frequency and particle concentration on droplet size, size uniformity and ejection velocity. In this work, Al/JP-10/OA nanofluids with aluminum mass fractions of 0.1 wt. %, 0.5 wt. % and 1.0 wt. % were tested under liquid flow rates of 1.1–1.5 mL/min and driving frequencies of 10–50 kHz, with measured droplet diameter ranging from 241.04 μm to 292.26 μm and ejection velocity ranging from 1.65 m/s to 2.45 m/s. The results demonstrate that average droplet diameter increases linearly with flow rate and decreases monotonically with driving frequency. Compared with the 0.1 wt. % nanofluid, the 1.0 wt. % nanofluid shows a 4.4% larger droplet diameter and 12.1% lower ejection velocity, while the 0.1 wt. % sample retains excellent monodispersity with a size *Span* below 0.098. The multi-scale regulation mechanisms involving viscous variation, shear-thinning rheology and particle agglomeration are further clarified. This study provides fundamental data and theoretical support for atomization design of nanofluid aviation fuels.

## 1. Introduction

The rapid development of hypersonic vehicles and cruise missiles has put forward extreme requirements for the energy density, combustion efficiency and dynamic response of aerospace power systems [1]. High-density hydrocarbon fuels, represented by JP-10 (exo-tetrahydrodicyclopentadiene), have become the most widely used aviation fuels due to their high volumetric heating value (39.6 MJ/L), excellent low-temperature fluidity and outstanding thermal stability [2,3]. However, as the performance boundaries of power systems continue to expand, the energy density and combustion rate of pure JP-10 have approached the theoretical limit of hydrocarbon fuels, which can hardly meet the demands of next-generation propulsion systems [4].

Doping high-activity nano-metal particles into hydrocarbon fuels to prepare nanofluid fuels is a promising technical path to break through fuel performance bottlenecks [5,6,7]. Aluminum nanoparticles feature high mass calorific value, strong reactivity and clean combustion products [8,9]. When doped into liquid fuels, they can simultaneously improve the volumetric energy density and combustion reaction rate of the system, significantly shorten the ignition delay period and enhance combustion heat release [10,11,12]. In recent years, Al/JP-10 nanofluid fuels have become a research hotspot in the field of energetic materials and propulsion technology [13,14].

Atomization and droplet formation is the primary step in the combustion process of liquid fuels [15]. The initial size, uniformity and velocity of droplets directly determine the evaporation rate, flame propagation characteristics and combustion stability. Compared with turbulent spray combustion in practical engineering, isolated combustion experiments with monodisperse droplets can decouple complex factors such as flow turbulence and inter-droplet interactions, and accurately obtain core parameters such as intrinsic evaporation rate constant, ignition delay time and micro-explosion characteristics [16]. Generating monodisperse droplets with uniform size, controllable velocity and good repeatability is the core prerequisite for high-precision single-droplet combustion experiments.

The incorporation of nanoparticles significantly alters the density, viscosity, surface tension and rheological properties of the fuel, and further modifies the whole process of jet instability, breakup and droplet formation. Current research on Al/JP-10 nanofluids mainly focuses on dispersion stability, evaporation, micro-explosion and combustion exothermic characteristics. Du et al. [17] systematically investigated the regulation mechanism of oleic acid dispersant on the stability of Al/JP-10 nanofluids and revealed the non-monotonic effect of particle concentration on dispersion stability. Dou et al. [18] verified through scramjet experiments that aluminum nanoparticles can significantly improve the combustion efficiency and thrust performance of JP-10 fuel. Paul et al. [19] studied the micro-explosion behavior of Al/JP-10 suspension fuels under high pressure and found that nanoparticles can significantly enhance the micro-explosion intensity of droplets. However, all the above studies take pre-generated droplets as research objects and pay little attention to the initial process of droplet formation induced by nano-doping.

In the field of monodisperse droplet generation technology, piezoelectric-driven drop-on-demand (DOD) technology has become the mainstream solution for single-droplet experiments due to its strong size controllability, high generation frequency and no additional airflow interference [10,20,21]. Single-chip piezoelectric actuators are widely used in high-frequency droplet generation scenarios owing to their simple structure, fast response and strong adaptability [22]. Zhu et al. [23] developed a nozzle-driven piezoelectric micro-jet device and achieved precise ejection of micro-droplets of high-viscosity fluids. Yang et al. [7] improved the stable jet frequency of piezoelectric nozzles to hundreds of kilohertz through multi-pulse waveform optimization. Nevertheless, existing studies mostly target civilian fields such as inkjet printing and biomanufacturing, and there is still a lack of systematic research on the droplet breakup law, stable operating boundary and physical property adaptation mechanism for multiphase aviation fuels containing nanoparticles.

In terms of droplet characterization, phase Doppler anemometry (PDA), based on the laser Doppler effect and light scattering phase difference principle, can synchronously measure droplet size and three-dimensional velocity in a non-contact manner, and is recognized as the standard method for atomization characterization [24]. Commercial PDA systems are mostly adapted to continuous spray fields, while customized measurement systems for high-frequency discrete droplets can further improve sampling efficiency and timing synchronization. Combined with high-speed microscopic imaging for morphological observation and result verification, complementary qualitative observation and quantitative measurement can be achieved, greatly improving the reliability of experimental data [25,26].

From the perspective of large-scale engineering applications, nano-aluminum has high procurement cost compared with submicron aluminum, which boasts mature production and lower prices for aviation fuel mass utilization. There is an evident performance trade-off: submicron aluminum has smaller specific surface area, slower heat release and worse suspension stability, yet it exhibits better anti-oxidation capacity and milder viscosity growth in JP-10, alleviating poor ejection velocity and monodispersity at high concentrations for large-flow DOD devices. This work focuses on nano-aluminum JP-10 droplet mechanisms, and future tests on mixed submicron/nano-aluminum fuels will be conducted to optimize formulations balancing cost, dispersion and combustion performance [27,28].

To sum up, the regulation mechanism of aluminum nanoparticle concentration on the single-droplet generation characteristics of JP-10-based fuels has not been revealed, and there is a lack of coupled impact analysis and in-depth mechanism explanation of operating parameters and physical property parameters. Aiming at this research gap, this work constructs a piezoelectric-driven monodisperse droplet generation platform combined with PDA and high-speed imaging, carries out experiments under multiple working conditions, and reveals the regulation mechanism of nano-doping on droplet generation from multi-scales. The main research contents include: (1) preparation and physical property characterization of Al/JP-10/OA nanofluids with different concentrations; (2) construction of the experimental system and clarification of measurement principles and data processing methods; (3) investigation of the effects of flow rate and driving frequency on droplet characteristics of nanofluids with different concentrations; and (4) in-depth revelation of the regulation mechanism of nanoparticle doping from multiple dimensions.

## 2. Materials and Methods

### 2.1. Materials and Nanofluid Preparation

JP-10, one of the jet fuels, was used as the base fuel. It was obtained from Tianjing University, Tianjin, China. Aluminum nanoparticles with a median diameter of 50 nm were selected as the dopant phase, commercially purchased from Dk Nano Technology Co., Ltd., Beijing, China. The surfactant oleic acid (OA, analytical grade) was used as the dispersant, which was purchased from Maclean Biochemical Technology Co., Ltd., Shanghai, China. To ensure dispersion stability, the mass ratio of aluminum nanoparticles to oleic acid was fixed at 1:2.5. Three mass fractions of aluminum nanoparticles, i.e., 0.1 wt. %, 0.5 wt. % and 1.0 wt. %, were set in the experiments.

All nanofluid samples were prepared via a two-step method. First, the weighed Al powder was put into the oleic acid and then oscillated in an ice bath (15 ± 5 °C) using an ultrasonic oscillator for 45 min, so that the oleic acid was fully wrapped onto the surface of the Al nanoparticles, to realize preliminary dispersion and surface modification. Second, the JP-10 was added into the mixtures of Al and oleic acid and then oscillated in the same temperature ice bath by the ultrasonic oscillator for 45 min to form uniformly dispersive suspensions.

To suppress aluminum nanoparticle oxidation, all samples were prepared in an ice bath at 15 ± 5 °C with a fixed Al-to-oleic acid mass ratio of 1:2.5. Oleic acid uniformly coats Al nanoparticles via coordination bonds to form a dense organic barrier, isolating dissolved oxygen in JP-10 and mitigating oxidation during preparation. All droplet tests were performed at room temperature and atmospheric pressure with freshly prepared fluids, and each test run lasted less than 30 min. Under low-temperature, short-duration conditions, oxidation is negligible and barely alters fluid thermophysical properties or droplet dynamics.

Nevertheless, severe oxidation occurs during long-term storage or high-temperature atomization/combustion, forming inert alumina shells on Al surfaces. Alumina weakens thermal conduction and energy release, while oxidized particles show enhanced van der Waals attraction, triggering massive agglomeration and destabilizing the nanofluid.

The basic physical properties of pure JP-10 and nanofluids were characterized at 25 ± 1 °C and atmospheric pressure. Density was measured by the standard density bottle method, dynamic viscosity by an Ostwald viscometer, and surface tension by the du Noüy ring method. The Ostwald viscometer has a capillary inner diameter of 0.6 mm and kinematic viscosity range of 0.5–10 mm^2^/s, accompanied by a thermostatic water bath with ±0.1 °C temperature precision. Each sample was measured five times at 25 °C for averaging. The du Noüy surface tension meter uses a 59.2 mm circumference Pt-Ir ring with 0.01 mN/m force resolution. The temperature-compensated module is applicable for liquids of 20–60 °C, with relative surface tension measurement error ≤0.5%. The ultrasonic oscillator works at 40 kHz with 400 W output power; it can stably maintain a dispersion temperature of 10–80 °C, and continuous ultrasonication can last for a maximum of 90 min. The results are listed in Table 1. With the increase in aluminum particle concentration, the fluid density and dynamic viscosity increase monotonically, while the surface tension shows a slight downward trend due to the surface activity of oleic acid, and the decline rate gradually slows down with the increase in concentration modulated by the interfacial adsorption of aluminum particles.

### 2.2. Experimental Setup

**Figure 1:**
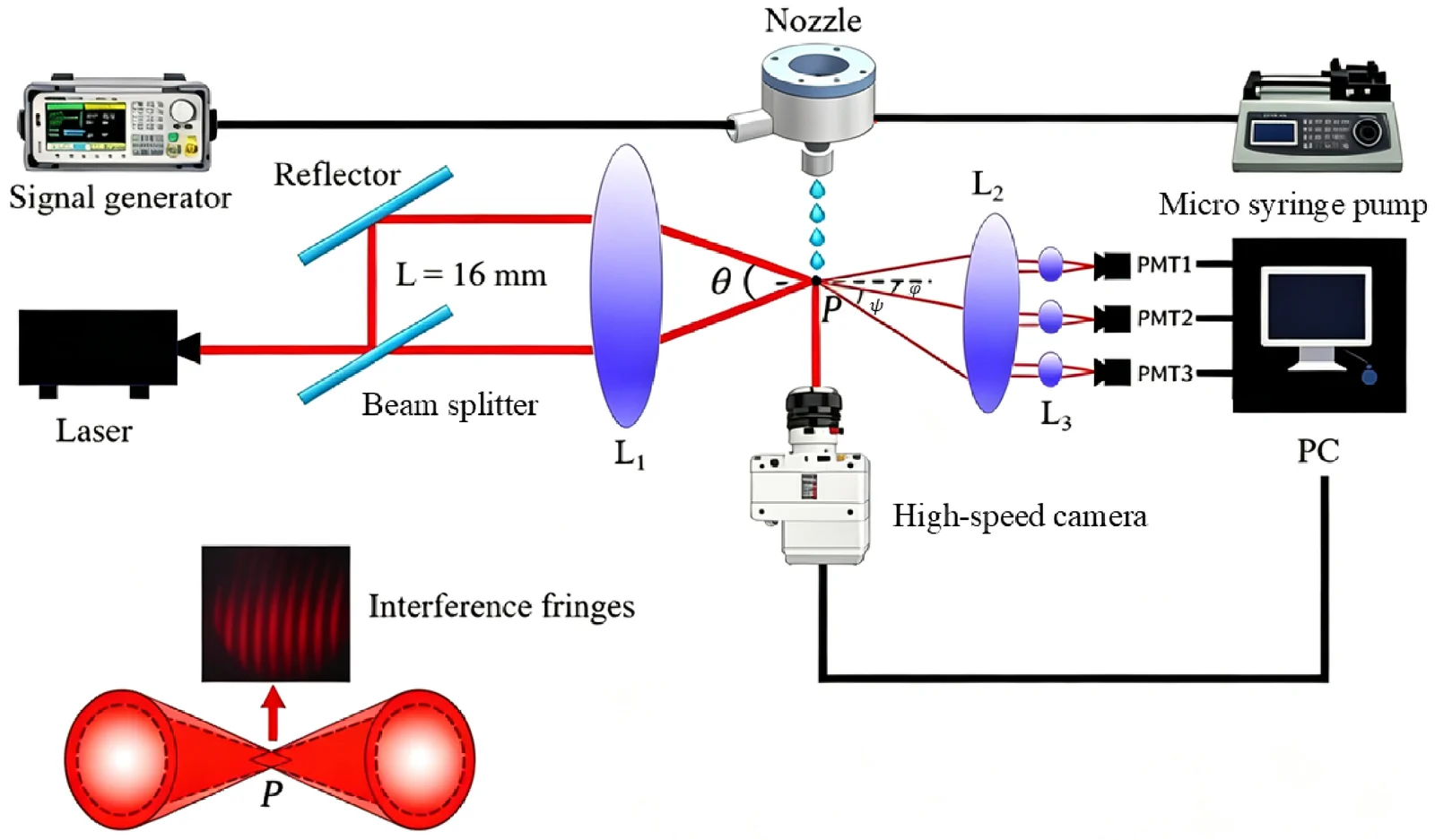
Schematic of experimental setup. (L_1_, focusing lens; L_2_, receiving lens; L_3_, relay lens).

To realize the synchronous, non-contact and high-precision measurement of droplet velocity and size under high-frequency conditions, the whole experimental system adopts a modular design, which consists of three parts: a piezoelectric-driven DOD droplet generator, a phase Doppler optical measurement unit and a high-speed camera observation module, as shown in Figure 1. The three devices are fixed in space and coincide in the measurement area, which enables simultaneous droplet generation, laser signal acquisition and high-speed image recording, ensuring the accuracy, reliability and repeatability of the experimental data.

#### 2.2.1. Piezoelectric DOD Droplet Generation Module

The piezoelectric droplet generation unit acts as the droplet supply module of the whole measurement system, which is designed to generate monodisperse droplets with good sphericity, uniform velocity and consistent size continuously and stably under high-frequency excitation. It is mainly composed of a micro syringe pump, a piezoelectric ceramic vibration assembly, a signal generator, a custom micro-orifice nozzle and fluid pipelines. The micro syringe pump provides stable and pulse-free volume flow with an adjustment range of 555.459 nL/min–83.318 mL/min and a control accuracy of ±0.001 mL/min. It ensures a stable liquid jet morphology at the nozzle outlet and avoids random variations in droplet velocity and size caused by flow fluctuations. The liquid output from the syringe pump enters the piezoelectric vibration module through a polytetrafluoroethylene (PTFE) flexible pipeline, forming a continuous and steady liquid jet.

The vibration core adopts a single-chip piezoelectric ceramic, which is firmly bonded to a brass vibrating plate with epoxy resin. Driven by the periodic square wave voltage output from the signal generator, the piezoelectric ceramic produces an inverse piezoelectric effect, generating high-frequency micro-amplitude bending vibration and transferring the vibration energy to the internal liquid. The vibration induces Rayleigh instability at the nozzle outlet, making the liquid jet break up uniformly at a fixed period and form regularly arranged monodisperse droplets. A stainless steel micro-orifice nozzle with a diameter of 100 μm fabricated by laser drilling was adopted. The driving frequency and voltage amplitude can be flexibly adjusted via the signal generator to adapt to droplet generation requirements under different working conditions. The driving voltage amplitude was fixed at 10 V in all experiments.

Assuming that the fluid medium is incompressible fluid, then according to the mass and volume conservation of the fluid, the theoretical droplet size of the droplet generated by the monodisperse droplet generator under ideal conditions can be obtained. The formula is as follows:

(1) $$d=\sqrt[{3}]{\frac{q}{600f\pi }}\times 10^{3}$$

where *d* is the theoretical particle size generated by the monodisperse droplet device, *f* is the piezoelectric driving frequency, and *q* is the flow rate.

#### 2.2.2. Phase Doppler Anemometry Module

The phase Doppler anemometry (PDA) module, based on the laser Doppler effect and light scattering phase difference principle, can synchronously measure the droplet size and axial velocity without contact. The whole module has a stable optical path and fast signal response, and is suitable for continuous dynamic detection of high-frequency discrete droplets. It consists of an incident optical subsystem and a collection optical subsystem. The incident optical subsystem is composed of a He-Ne laser, a beam splitter, total reflection mirrors and a focusing lens L_1_ with a focal length *f*_1_ = 200 mm. The laser model is HNL-100R (L) with an output wavelength of 633 nm, TEM_00_ fundamental mode, minimum output power of 10 mW and polarization ratio > 1000:1. The laser beam is split into two parallel beams with a separation of 16 mm by the beam splitter and reflection mirrors, and then focused by the lens to form an ellipsoidal measurement volume at point *P*. After calibration, the measurement volume has a height of 80.6 μm, a width of 80.7 μm, a length of 1.76 mm and an effective measurement volume of about 6.09 mm^3^.

The collection optical subsystem adopts a forward scattering layout with a scattering angle *φ* = 30°. It consists of one receiving lens L_2_ with *f*_2_ = 150 mm focal length, three relay lenses L_3_ with *f*_3_ = 30 mm focal length and three photomultiplier tubes (PMTs). The PMT model is H10720-110, with a spectral response range of 230–700 nm and a response frequency up to 10 MHz, which can realize efficient photoelectric conversion of weak scattered light signals. After photoelectric conversion by the PMTs, the electrical signals are collected by a high-speed data acquisition card (model JY9813) with a maximum sampling rate of 20 MS/s and an analog input bandwidth of 84 MHz. A Doppler signal visualization processing program was developed on the Visual Studio platform using C# language, which integrates signal filtering, spectrum analysis and characteristic parameter calculation. The measurement point is located 10 mm below the nozzle outlet to ensure that droplets have completed breakup and entered a stable motion stage.

#### 2.2.3. High-Speed Imaging Module

The high-speed imaging module consists of a high-speed camera (Phantom VEO E310L, Vision Research, Inc., Wayne, NJ, USA), a Navitar zoom lens and an LED illumination source (Constellation 120E, Veritas, Inc., Pasadena, CA, USA). The shooting direction is perpendicular to the droplet trajectory, and the shooting area coincides spatially with the PDA measurement volume. The shooting frame rate was set to 10,000 fps in the experiment. To ensure illumination uniformity, a parallel LED backlight source was used to illuminate the measurement area, so that droplets present high-contrast contours in the image, effectively suppressing specular reflection and ambient stray light interference. High-speed images are mainly used for droplet morphology observation, breakup process analysis, and calibration and cross-validation of PDA measurement results.

### 2.3. Image and Doppler Signal Processing

#### 2.3.1. High-Speed Image Processing

A systematic high-speed image processing procedure was performed on the raw droplet images: first, the background regions at the top and bottom 30% of the image height were cropped to reduce computation and exclude edge interference; then, adaptive threshold binarization was applied to make the droplet region black and the background white; subsequently, morphological hole filling was performed to fill the internal blank area of droplets caused by uneven illumination, obtaining complete binary droplet contours. Finally, the pixel area and perimeter of droplets were counted based on the complete contours, and the actual geometric dimensions were converted according to the equivalent diameter formula and calibration coefficient, which were used to cross-validate with PDA data.

The equivalent droplet diameter calculated by the area-perimeter method is expressed as: *d* = 4*S*/*L*, where *d* is the equivalent droplet diameter, *S* is the projected area, and *L* is the contour perimeter. The droplet axial velocity calculated from consecutive frames is given by: *v* = kΔ*x*_pix_/Δ*t*, where k is the image calibration coefficient (μm/pixel), Δ*x*_pix_ is the pixel displacement of the droplet centroid between adjacent frames, and Δ*t* is the time interval between frames determined by the shooting frame rate.

The size span is adopted to characterize the monodispersity of droplets, defined as: *Span* = (*D*_90_ − *D*_10_)/*D*_50_ where *D*_10_, *D*_50_ and *D*_90_ are the 10th, 50th and 90th percentiles of the cumulative size distribution, respectively. A smaller *Span* value indicates a narrower size distribution and better monodispersity. Generally, *Span* < 0.15 is regarded as good monodispersity. No less than 400 valid droplets were counted for each working condition to ensure statistical significance.

#### 2.3.2. PDA Measurement Principle and Signal Processing

The core principle of phase Doppler anemometry includes two aspects: the Doppler shift induced by droplet motion is quantitatively related to the moving velocity, and the phase difference in scattered light at different receiving positions is linearly proportional to the droplet diameter. The quantitative relationship between droplet axial velocity and Doppler shift is [29]:

(2) $$U=\frac{f_{d}\lambda }{2\sin\left(\theta /2\right)}$$

where *U* is the axial velocity of the droplet, *f_d_* is the Doppler shift, *λ* is the wavelength of the incident laser, and *θ* is the intersection angle of the two incident beams.

The relationship between droplet diameter *D* and scattered light phase difference Δ*Φ* is [29]:

(3) $$D=\Delta \phi \frac{\lambda \sqrt{2\left(1+\cos\theta \cos\varphi \cos\psi \right)\left[1+n^{2}-n\sqrt{2\left(1+\cos\theta \cos\varphi \cos\psi \right)}\right]}}{-2\pi \cdot n\cdot \sin\psi \sin\theta }$$

where *D* is the droplet diameter based on PDA measurement, Δ*Φ* is the phase difference in scattered light between two receiving channels, *φ* is the forward scattering angle, *ψ* is the intersection angle of scattered beams, and *n* is the refractive index of the droplet.

In Formulas (2) and (3), an averaged effective refractive index *n* is adopted for droplet-size inversion. This representative refractive index is experimentally measured at the experimental temperature and the 633 nm wavelength of the He-Ne laser equipped in the PDA system, covering Al/JP-10/OA nanofluids across the entire tested concentration range. Within the aluminum particle loading of 0.1–1.0 wt. %, low-content nanoparticles and oleic acid surfactant only produce slight fluctuations in the refractive index of the liquid phase. Such minor discrepancies among different samples exert nearly no influence on PDA diameter retrieval based on Mie scattering.

**Figure 2:**
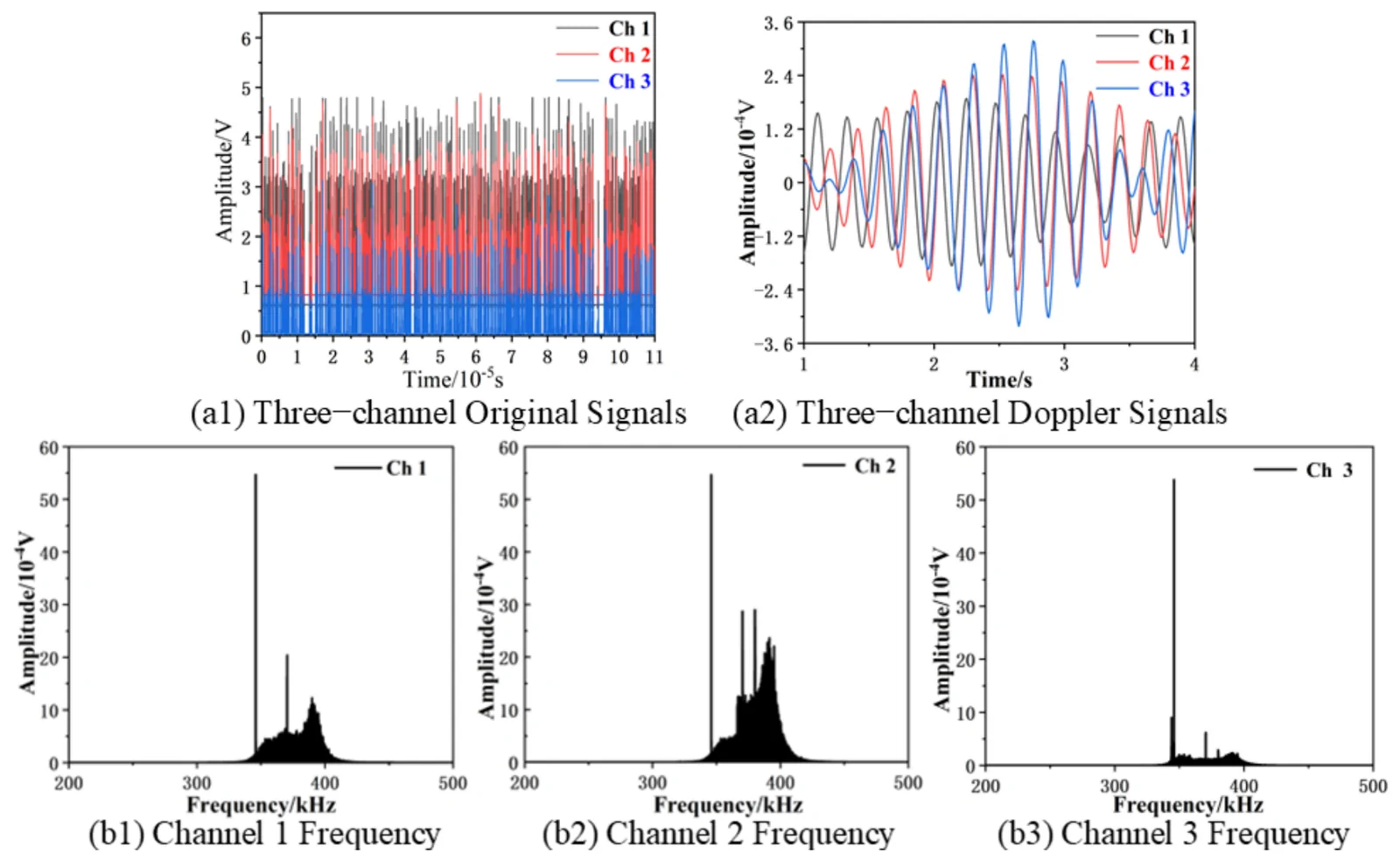
The signal processing of PDA measurement for Doppler frequency ((**a1**,**a2**): three-channel time-domain Doppler signals; (**b1**–**b3**): three-channel frequency-domain Doppler signals).

To accurately extract the Doppler frequency and phase information from particle scattered light, a complete signal processing workflow of “infinite impulse response (IIR) high-pass filtering + finite impulse response (FIR) band-pass filtering + fast Fourier transform (FFT) spectrum and phase analysis” was adopted. The raw photoelectric signal usually contains DC offset, low-frequency noise and ambient vibration interference, which will mask the high-frequency Doppler signal (Figure 2(a1,a2)). An IIR high-pass filter is first used to remove low-frequency components and highlight the effective Doppler signal. And then, FIR band-pass filtering is further performed to extract the target Doppler frequency band. The FIR filter has strictly linear phase characteristics, which ensures no phase distortion during signal filtering and guarantees the accuracy of subsequent droplet size calculation. FFT is performed on the filtered pure Doppler signal to convert the time-domain signal into the frequency domain (Figure 2(b1–b3)). The peak Doppler frequency is directly obtained, and the phase values of each channel are extracted from the complex spectrum. Finally, the size and velocity of each droplet are calculated.

Using ethanol as the calibration medium at a fixed driving frequency of 30 kHz, the comparison between the high-speed camera calibration results and PDA measurement results under different syringe pump flow rates is presented.

Ethanol was selected as the calibration medium at the fixed driving frequency of 30 kHz for three reasons: First, ethanol is a homogeneous single-phase liquid without solid particles, which eliminates interference from particle agglomeration and uneven physical properties during calibration. Second, the viscosity and surface tension of ethanol at 25 °C are close to those of pure JP-10, leading to similar jet breakup morphology and spherical droplet characteristics, which guarantees consistent calibration conditions with formal nanofluid tests. Third, 30 kHz is the benchmark working frequency for all flow-variable experiments; calibrating under this condition ensures uniform measurement accuracy across all operating conditions. In addition, ethanol has moderate volatility and will not cause residual contamination inside the micro-nozzle.

It can be verified that the droplet size and velocity data acquired by the two methods are in high overall agreement. For droplet diameter measurement, the relative error is below 1.9%. While for droplet velocity measurement, the relative error is smaller than 2.1%. These quantitative results fully verify the reliability of the self-built PDA measurement system.

### 2.4. Experimental Design

All experiments were conducted under room temperature (25 ± 1 °C) and atmospheric pressure with a fixed nozzle diameter of 100 μm. Two groups of controlled variable experiments were designed: (1) Flow rate effect experiment: the piezoelectric driving frequency was fixed at 30 kHz, and the liquid flow rate was set to 1.1, 1.2, 1.3, 1.4 and 1.5 mL/min, with 5 working conditions in total. (2) Driving frequency effect experiment: the liquid flow rate was fixed at 1.3 mL/min, and the driving frequency was set to 10, 20, 30, 40 and 50 kHz, with 5 working conditions in total. Each group of experiments included three nanofluid fuels: 0.1 wt. % Al/JP-10/OA, 0.5 wt. % Al/JP-10/OA and 1.0 wt. % Al/JP-10/OA. All working conditions were repeated three times, and the average value was taken as the final result. The error bars in the figures represent the standard deviation of the three repeated experiments.

## 3. Results and Discussion

### 3.1. Effect of Liquid Flow Rate on Droplet Generation Characteristics

#### 3.1.1. Droplet Size and Size Distribution Under Different Liquid Flow Rate

**Figure 3:**
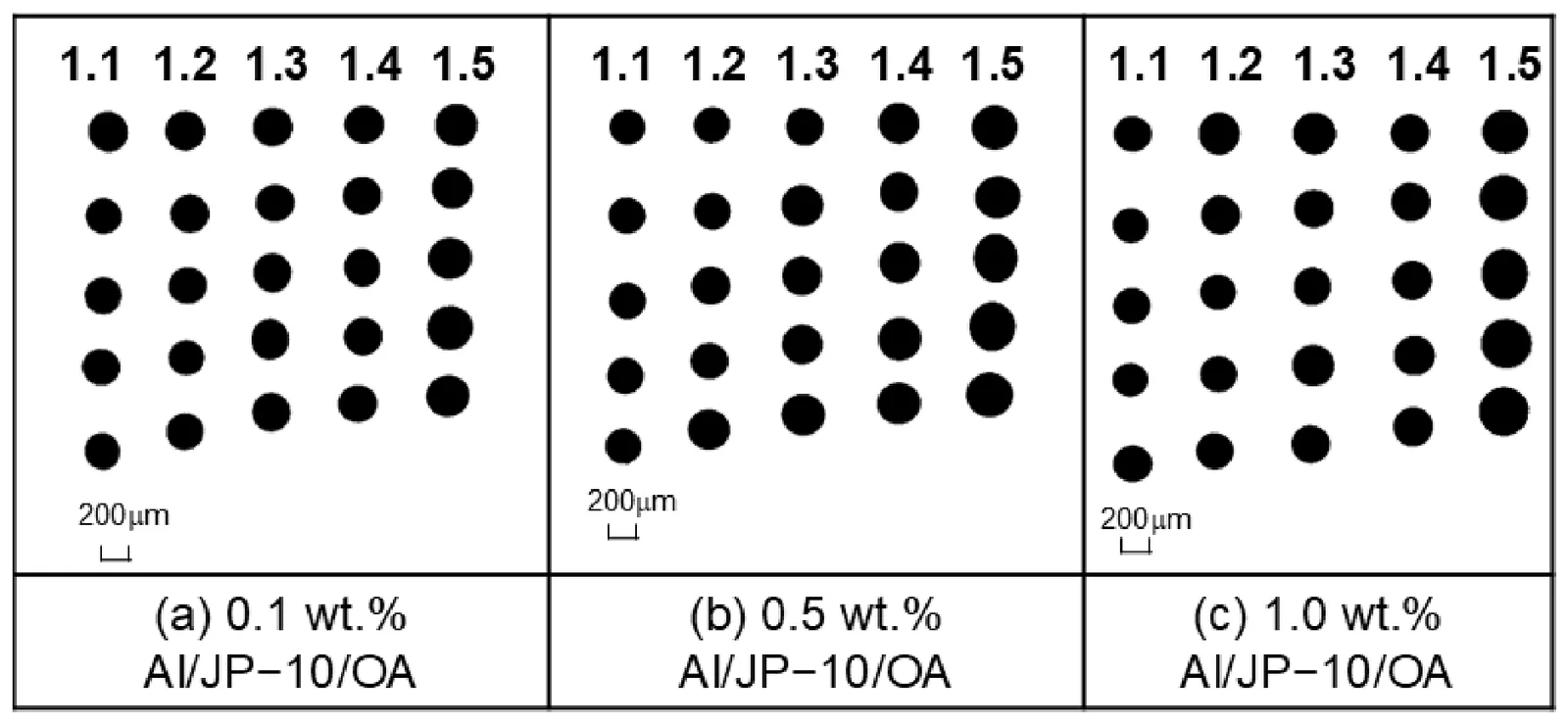
The droplet generation morphologies at representative flow rates (1.1, 1.2, 1.3, 1.4, 1.5 mL/min) of 0.1 wt. %Al/JP-10/OA (**a**), 0.5 wt. %Al/JP-10/OA (**b**), and 1.0 wt. %Al/JP-10/OA (**c**).

**Figure 4:**
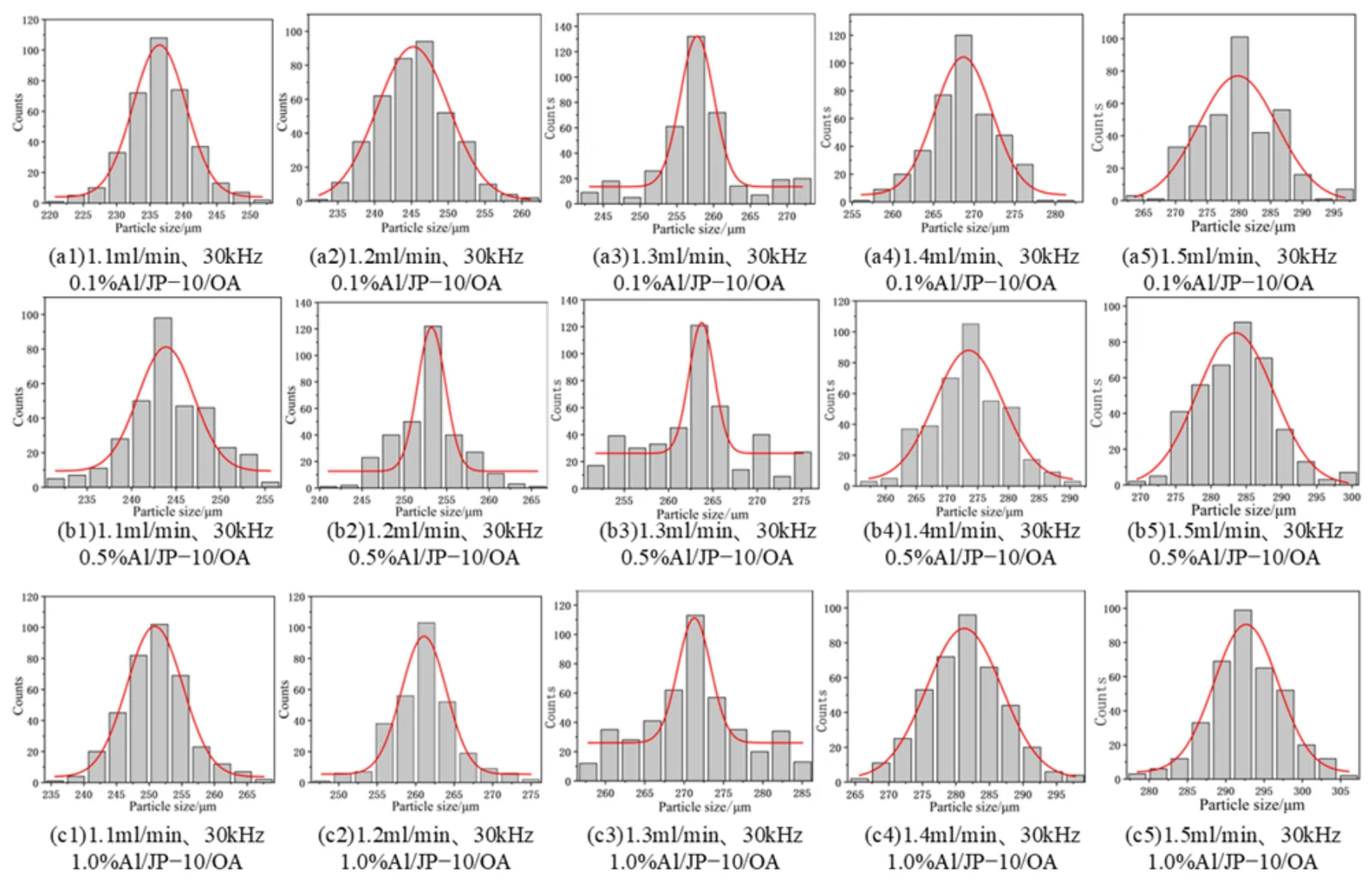
The corresponding droplet size distribution histograms at representative flow rates 0.1 wt. %Al/JP-10/OA (**a1**–**a5**), 0.5 wt. %Al/JP-10/OA (**b1**–**b5**), and 1.0 wt. %Al/JP-10/OA (**c1**–**c5**). (Red lines represent the Gaussian fitting curves of the data).

**Figure 5:**
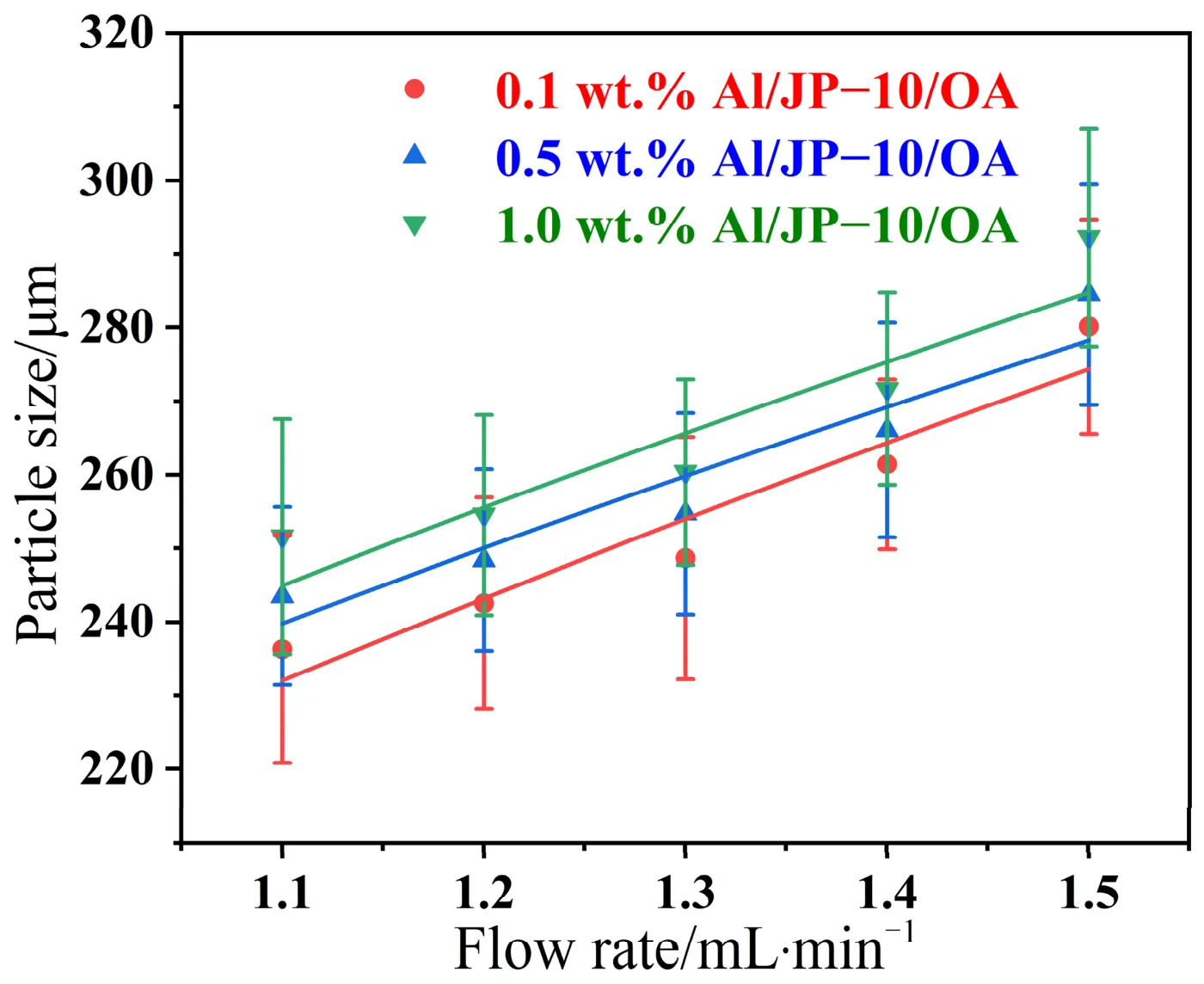
The variation curve of average droplet diameter across the entire flow rate range from 1.1 to 1.5 mL/min.

Figure 3 displays the droplet generation morphologies at the full experimental range of 1.1–1.5 mL/min. Figure 4 presents the corresponding droplet size distribution histograms for these three typical operating conditions, while Figure 5 plots the variation curve of average droplet diameter across the entire flow rate range from 1.1 to 1.5 mL/min.

At low flow rates, nanofluids of all concentrations produce discrete droplets with regular morphology and clear boundaries, with no obvious satellite droplets or coalescence. As the flow rate increases, the droplet size grows markedly. Quantitative statistics over the full 1.1–1.5 mL/min range show that the average droplet diameter of all nanofluid samples increases nonlinearly and monotonically with rising flow rate. The underlying physical mechanism is that when the driving frequency is fixed, the droplet generation frequency remains constant. The total liquid volume passing through the nozzle increases nonlinearly with the flow rate per unit time, so the liquid volume allocated to each individual droplet increases synchronously, resulting in a power-law increase in the droplet diameter which satisfies the scaling relationship $d\propto q^{1/3}$ [30].

By comparing nanofluid fuels with different concentrations, it can be found that under the same flow rate, the higher the Al concentration, the larger the droplet size and the faster the growth rate of diameter with flow rate. Taking the 1.5 mL/min condition as an example, the average droplet diameter of 0.5 wt. % and 1.0 wt. % nanofluids are 284.48 μm and 292.26 μm, corresponding to increases of 1.6% and 4.4% compared with 0.1 wt. % nanofluids, respectively. The core reason for this phenomenon is that the doping of nanoparticles improves the overall viscosity and density of the fluid (seeing Table 1). On the one hand, the increased viscous force raises the jet breakup resistance, slows down the neck contraction of the liquid jet, increases the breakup difficulty, and thus enlarges the liquid volume entrained in a single breakup [31]. On the other hand, the increased density enhances the jet inertia, making it more difficult for gas–liquid interface disturbances to tear the jet, which finally manifests as larger initial droplet size and stronger flow rate sensitivity. In terms of size distribution, all fuels present a narrow-peak normal distribution at low flow rates, with *Span* values below 0.13 and good monodispersity. As the flow rate continues to rise, the fluid inertia force keeps increasing, and the piezoelectric vibration can hardly achieve precise and uniform cutting of the jet. The randomness of droplet breakup is enhanced, and coalesced droplets, tiny satellite droplets and irregular broken droplets are prone to occur, resulting in a significant increase in size standard deviation and distribution span. Among them, high-concentration nanofluids are more significantly affected by viscosity increase. Internal shear inhomogeneity and particle effects will further aggravate the breakup instability. Therefore, under high-flow-rate conditions, droplet distortion and coalescence are more prominent, and the monodispersity degradation is much more serious than that of low-concentration nanofluids [32]. At 1.5 mL/min, the *Span* value of 1.0 wt. % nanofluid rises to 0.20, losing good monodispersity.

#### 3.1.2. Droplet Ejection Velocity Under Different Liquid Flow Rate

**Figure 6:**
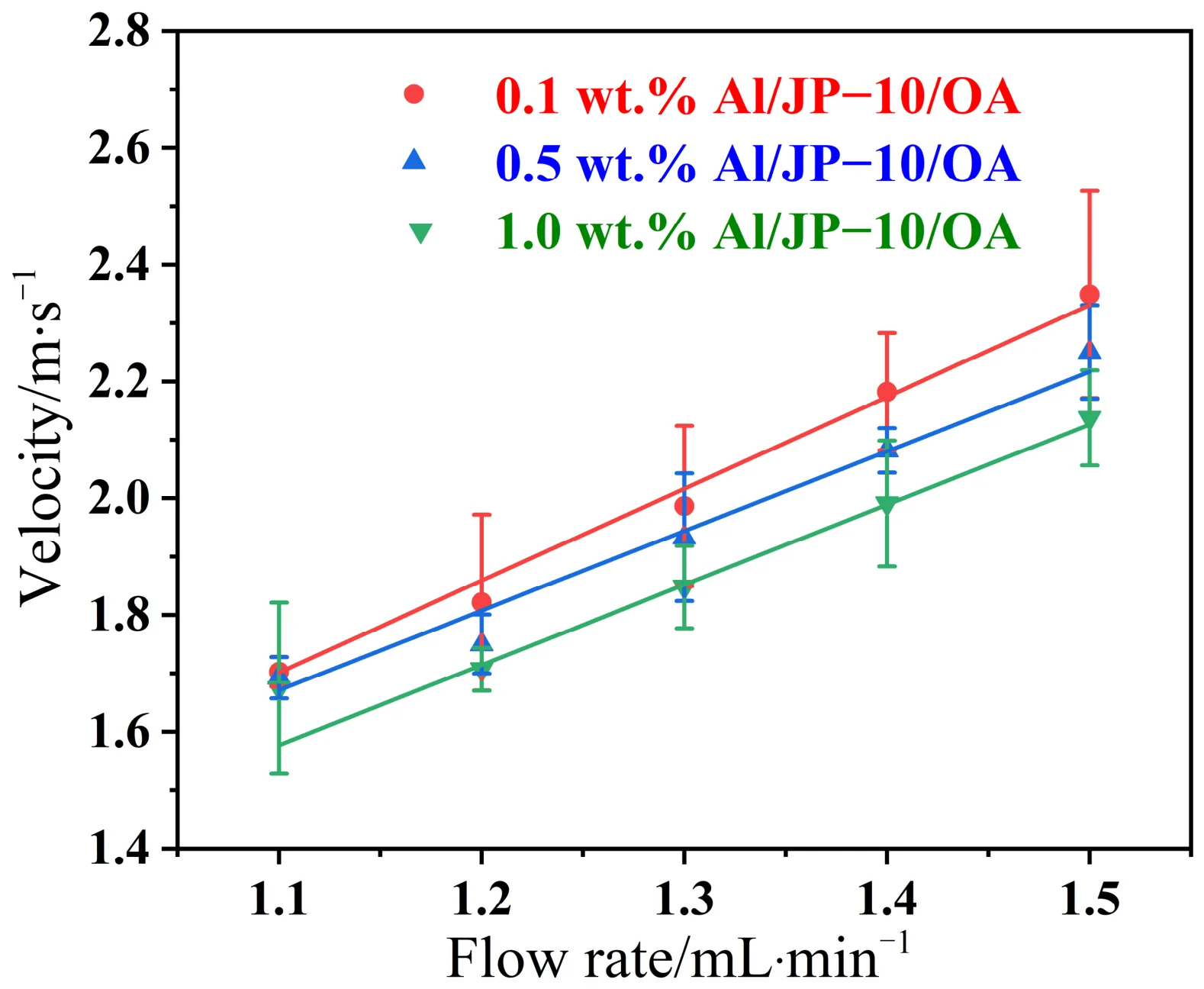
The variation in droplet ejection velocity across the entire flow rate range from 1.1 to 1.5 mL/min.

Figure 6 shows the variation in droplet ejection velocity with liquid flow rate. The average ejection velocity of all fuels increases approximately linearly with rising flow rate. The internal mechanism is that under a constant driving frequency, the increase in flow rate directly enlarges the pressure difference across the nozzle, significantly raising the axial velocity of the liquid jet at the nozzle outlet. Droplets inherit the axial kinetic energy of the jet after breakup, so the initial ejection velocity increases with flow rate. Meanwhile, the rise in flow rate moves the jet breakup position downstream, allowing droplets to obtain a longer acceleration distance before breakup, which further promotes the ejection velocity [33]. When compared at the same flow rate, the droplet ejection velocity exhibits a monotonically decreasing trend with rising aluminum nanoparticle concentration. At the representative flow rate of 1.3 mL/min, the 0.1 wt. % Al/JP-10/OA nanofluid yields a droplet ejection velocity of 1.99 m/s. As the particle concentration increases to 0.5 wt. % and 1.0 wt. %, the ejection velocity drops to 1.79 m/s and 1.75 m/s, respectively. Specifically, the 1.0 wt. % high-concentration nanofluid records a 12.4% velocity reduction relative to the 0.1 wt. % low-concentration sample. The reason is that high-concentration fluids have larger viscous damping, leading to more flow dissipation in the nozzle and liquid chamber, and a lower proportion of energy converted into droplet kinetic energy under the same pressure difference. Meanwhile, the increased density also requires more energy to obtain the same kinetic energy per unit volume of fluid. Multiple factors jointly lead to the decrease in ejection velocity [34].

### 3.2. Effect of Piezoelectric Driving Frequency on Droplet Generation Characteristics

#### 3.2.1. Droplet Size and Size Distribution Under Different Piezoelectric Driving Frequency

**Figure 7:**
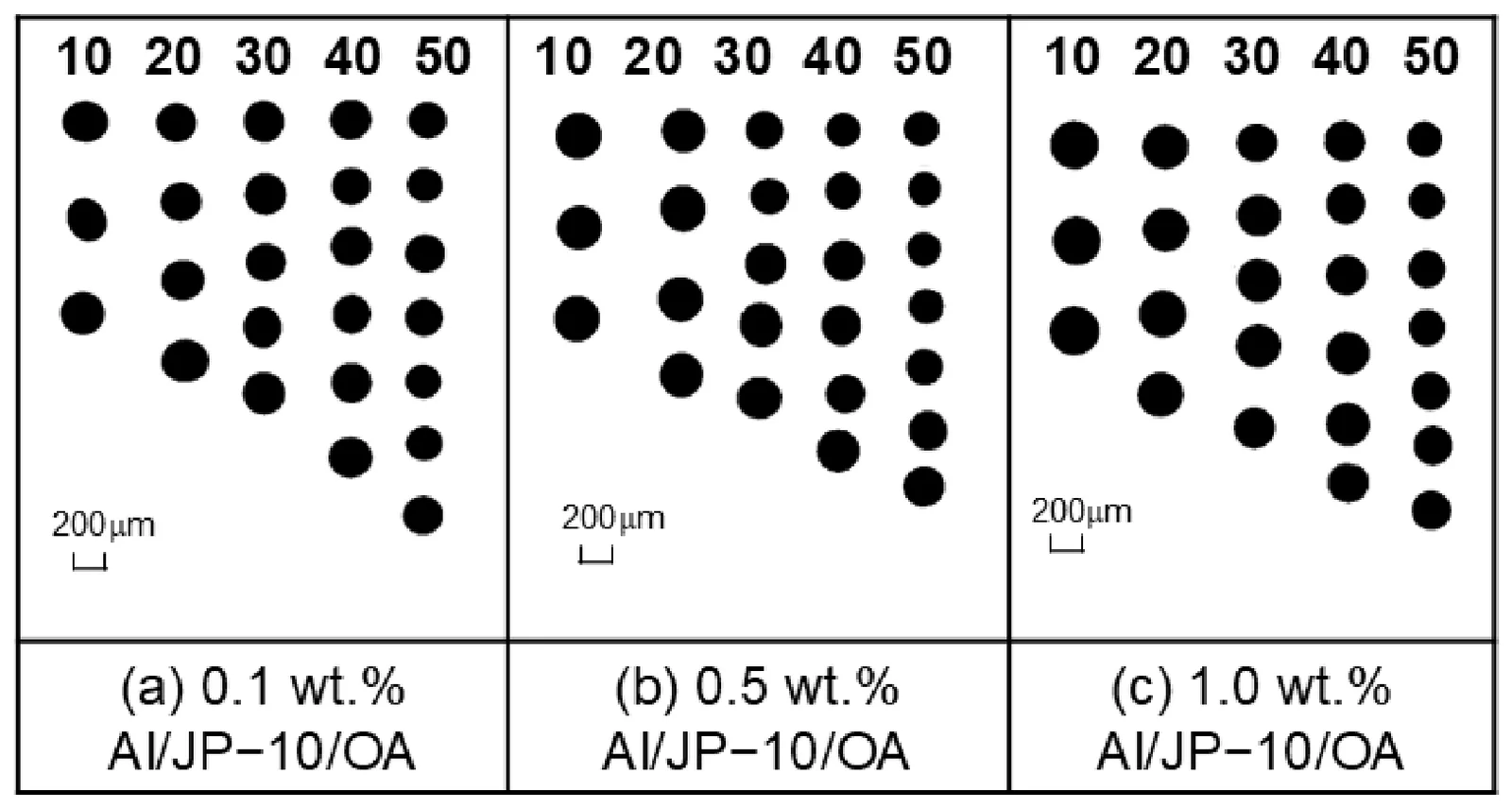
The droplet generation morphologies at three representative driving frequencies: 0.1 wt. %Al/JP-10/OA (**a**), 0.5 wt. %Al/JP-10/OA (**b**), and 1.0 wt. %Al/JP-10/OA (**c**).

**Figure 8:**
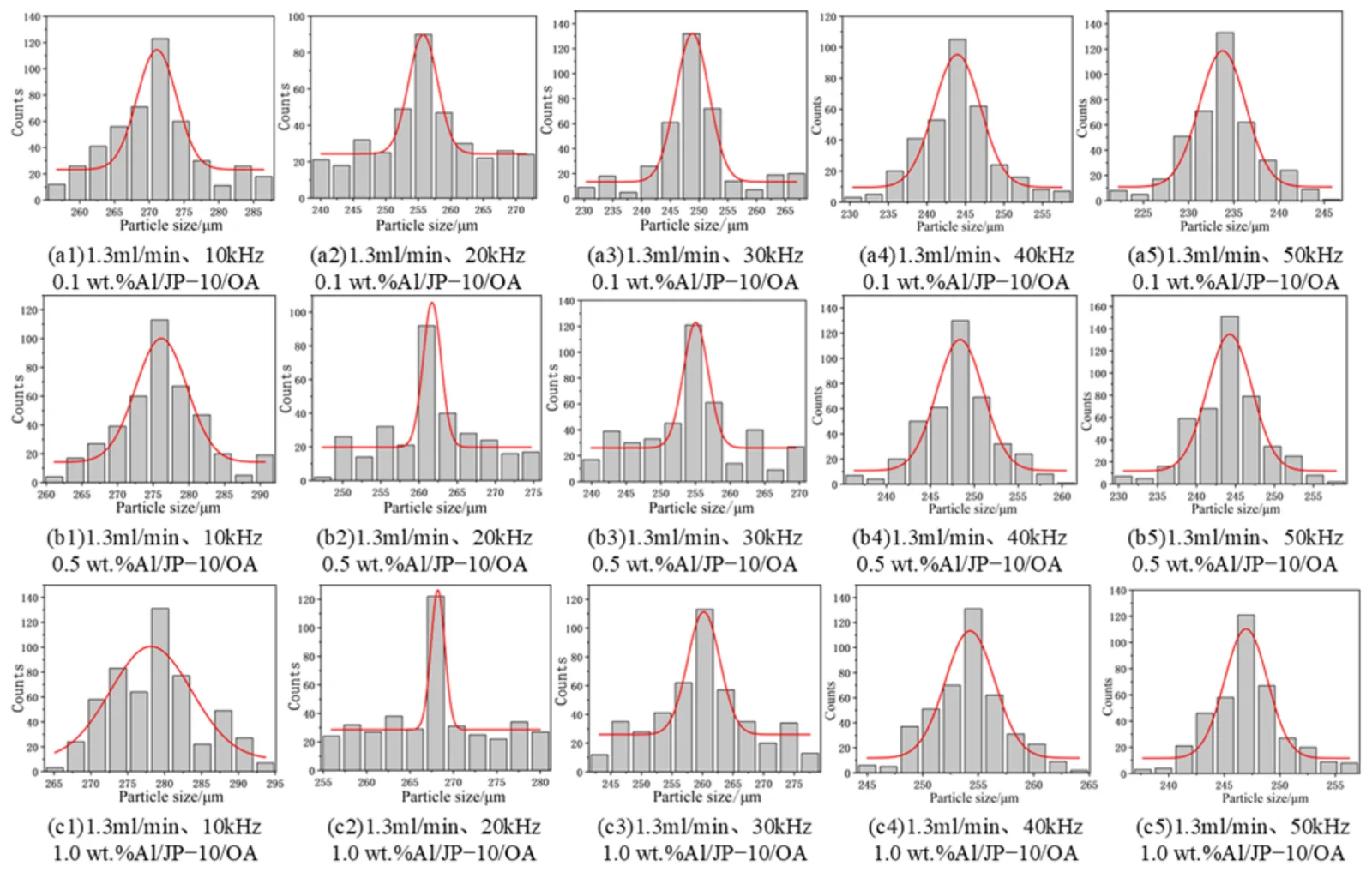
The corresponding droplet size distribution histograms at three representative driving frequencies: 0.1 wt. %Al/JP-10/OA (**a1**–**a5**), 0.5 wt. %Al/JP-10/OA (**b1**–**b5**), and 1.0 wt. %Al/JP-10/OA (**c1**–**c5**). (Red lines represent the Gaussian fitting curves of the data).

**Figure 9:**
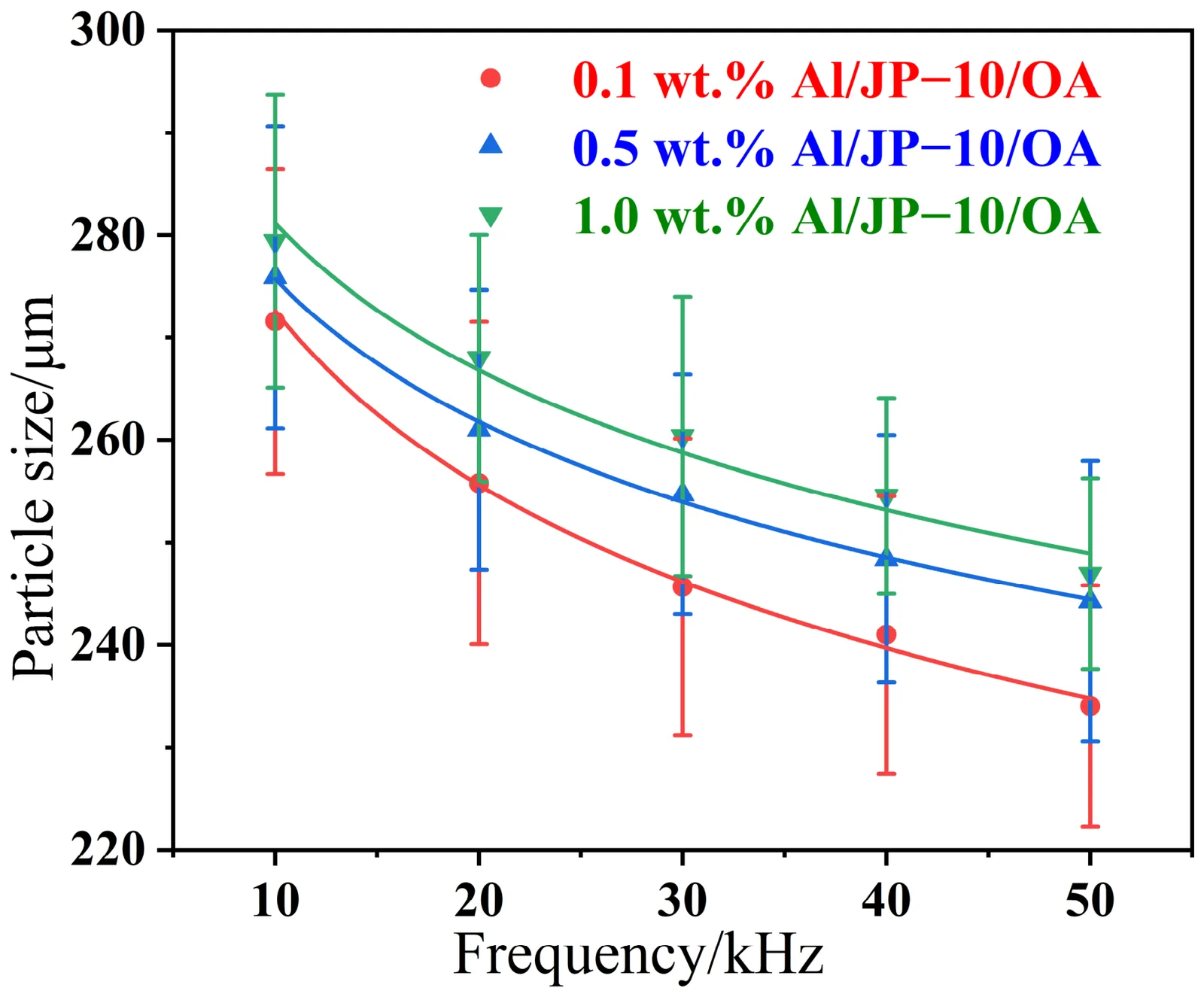
The variation in average droplet diameter across the entire driving frequency range from 10 to 50 kHz.

Figure 7 exhibits the droplet morphologies at the full experimental range of 10–50 kHz. Figure 8 presents the corresponding droplet size distribution histograms for these three typical operating conditions, while Figure 9 illustrates the variation trend of average droplet diameter across the entire frequency range from 10 to 50 kHz.

Within the full 10–50 kHz frequency band, all nanofluid samples can form clear, independent monodisperse droplets without obvious large-scale droplet coalescence, which verifies the operational stability of the piezoelectric generation system in this frequency range. As the driving frequency increases, the droplet diameter decreases visibly. At any identical driving frequency, the droplet size rises with increasing aluminum nanoparticle concentration, which is consistent with the statistical results.

Quantitatively, the average droplet diameter of all nanofluid samples decreases monotonically with rising driving frequency, following a theoretical power-law scaling of $d\propto f^{-1/3}$. Under a fixed liquid flow rate, a higher driving frequency means the liquid jet is cut more times per unit time by piezoelectric vibration, and the liquid volume allocated to each single breakup event becomes smaller, leading to a reduced droplet diameter. As the driving frequency increases from 10 kHz to 50 kHz, the average droplet diameter of the 0.1 wt. % nanofluid decreases from 271.59 μm to 241.04 μm, corresponding to a reduction of 11.2%; for the 1.0 wt. % nanofluid, the diameter drops from 275.89 μm to 248.27 μm, with a reduction of 10.0%. It can be found that the reduction amplitude of droplet size with frequency becomes smaller as the nanofluid concentration increases. This is because the introduction of nanoparticles elevates the fluid viscosity and jet breakup resistance, weakening the droplet refinement effect of high-frequency vibration. Meanwhile, high-viscosity fluids have a slower pressure propagation speed, and the liquid chamber pressure cannot fully recover under high-frequency driving, which increases the fluctuation of single ejection volume and reduces the regulation efficiency of driving frequency on droplet size [35].

Regarding size distribution, all nanofluid samples follow a normal distribution at each tested frequency. When the frequency rises from 10 kHz to 30 kHz, the distribution peak shifts toward smaller sizes, the overall distribution range narrows, the *Span* value decreases gradually, and the size uniformity is improved. When the frequency exceeds 40 kHz, the *Span* value of high-concentration nanofluids begins to rise, indicating degraded monodispersity. This arises from the insufficient droplet breakup of high-viscosity fluids at high frequencies, which is usually accompanied by the generation of satellite droplets and thus broadens the size distribution.

#### 3.2.2. Droplet Ejection Velocity Under Different Piezoelectric Driving Frequency

**Figure 10:**
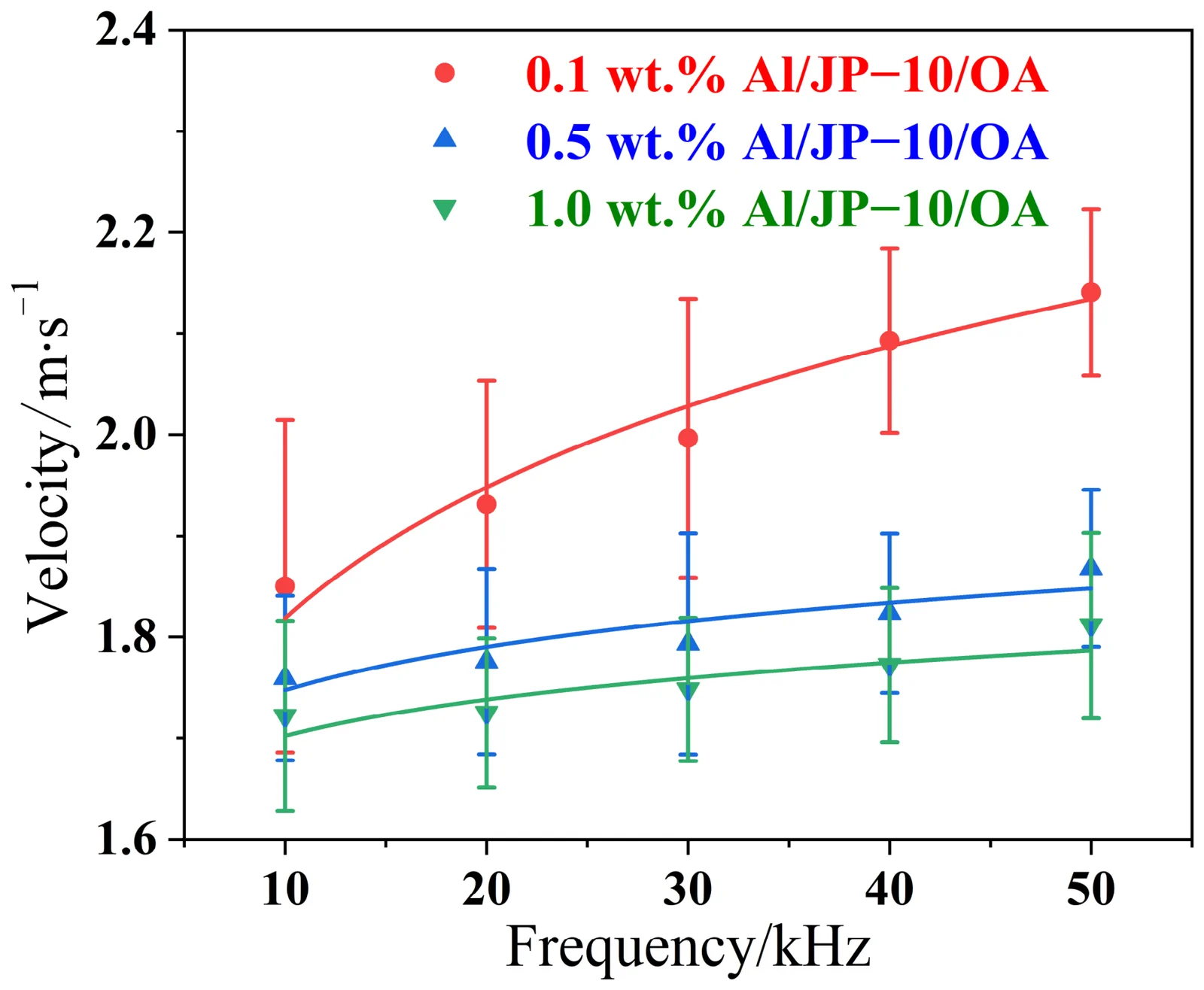
The variation in droplet ejection velocity across the entire driving frequency range from 10 to 50 kHz.

Figure 10 depicts the variation in droplet ejection velocity with driving frequency for nanofluid fuels with different aluminum concentrations. The ejection velocity of all nanofluid samples rises with increasing driving frequency, while the growth rate slows down gradually. The high-frequency vibration of piezoelectric ceramics applies instantaneous pulse kinetic energy to the liquid jet. The higher the driving frequency, the more pulse energy the liquid acquires per unit time, and the stronger the axial excitation received by droplets at the moment of breakup, which accordingly raises the initial ejection velocity. In the range of 10–30 kHz, the velocity increases rapidly; when the frequency exceeds 30 kHz, the growth trend moderates and a saturation effect appears. This is attributed to the insufficient pressure recovery time of the liquid chamber at high frequencies, which reduces the work efficiency of piezoelectric deformation, and further frequency increase cannot deliver more kinetic energy to the liquid.

Droplet ejection velocity decreases monotonically with rising nanoparticle concentration, and the velocity saturation point emerges earlier for higher-concentration nanofluids. The velocity of the 1.0 wt. % nanofluid levels off at approximately 30 kHz, whereas the 0.1 wt. % nanofluid maintains a noticeable growth trend even at 50 kHz. This further verifies the inhibitory effect of viscous damping on droplet acceleration: fluids with higher viscosity induced by heavier particle loading exhibit faster energy dissipation and lower energy utilization efficiency under high-frequency driving.

### 3.3. Underlying Regulation Mechanisms of Aluminum Nanoparticle Doping

#### 3.3.1. Macroscopic Force Balance Based on Dimensionless Analysis

**Figure 11:**
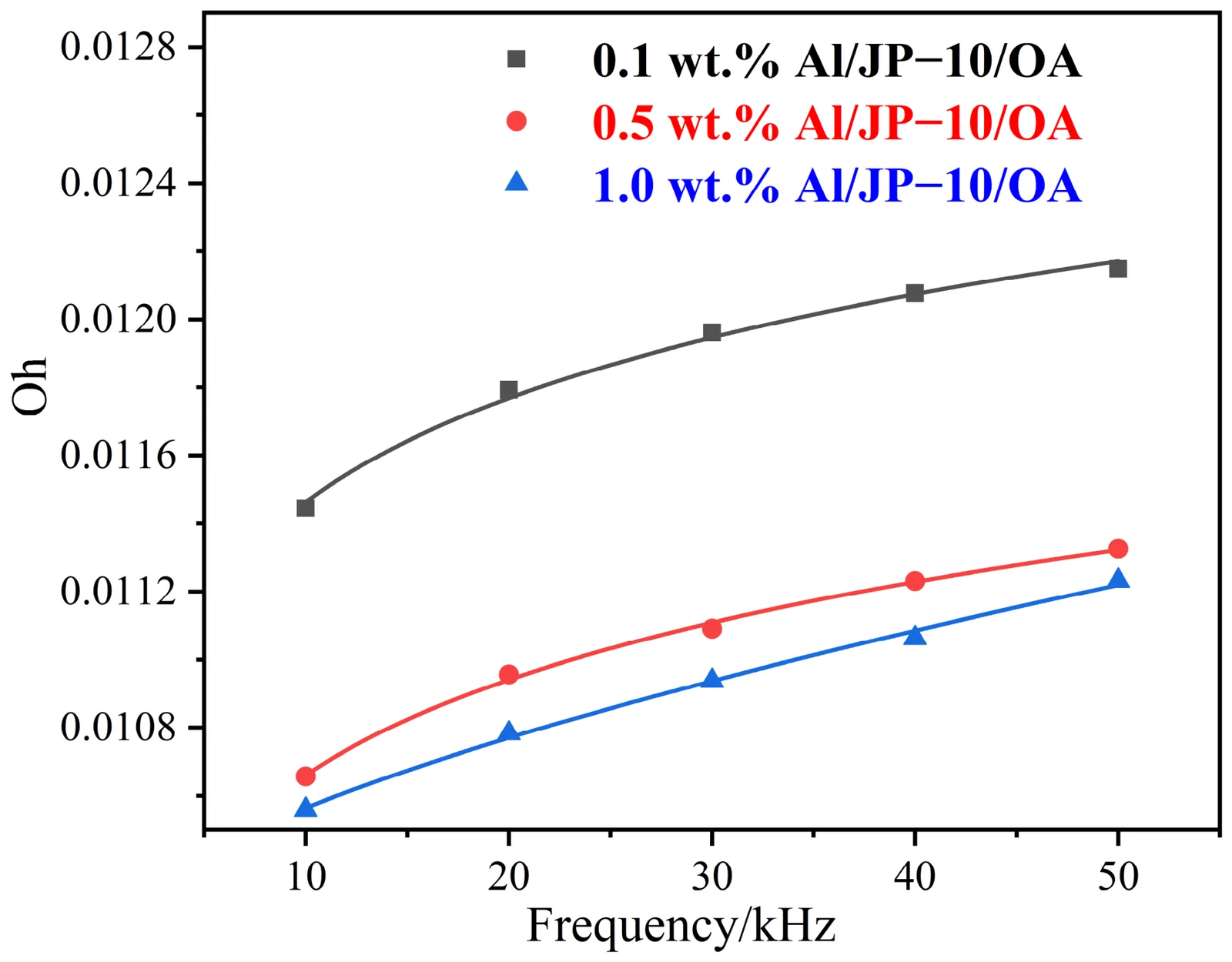
The *Oh* number of nanofluid droplets at different frequencies at 1.3 mL/min.

Droplet formation is essentially a process of Rayleigh instability and breakup of a liquid jet under external disturbances, and the final droplet size is determined by the coupled balance of inertial force, viscous force and surface tension. Aluminum particle doping directly changes the relative magnitude and balance of the three forces by modifying three macroscopic physical properties: density, viscosity and surface tension. According to the linear instability theory of viscous liquid jets, the Ohnesorge number (*Oh*) is the core dimensionless number characterizing jet breakup characteristics, which is defined as the ratio of viscous force to the resultant force of surface tension and inertial force [36]:

(4) $$Oh(\varphi )=\frac{\mu_{nf}(\varphi )}{\sqrt{\rho_{nf}(\varphi )\gamma_{nf}(\varphi )D}}$$

where *μ_nf_* is dynamic viscosity, *ρ_nf_* is density, *D* is droplet diameter, and *γ_nf_* is surface tension, respectively. The *Oh* number of each fuel at different frequencies at the flow rate of 1.3 mL/min are calculated and shown in Figure 11.

**Figure 12:**
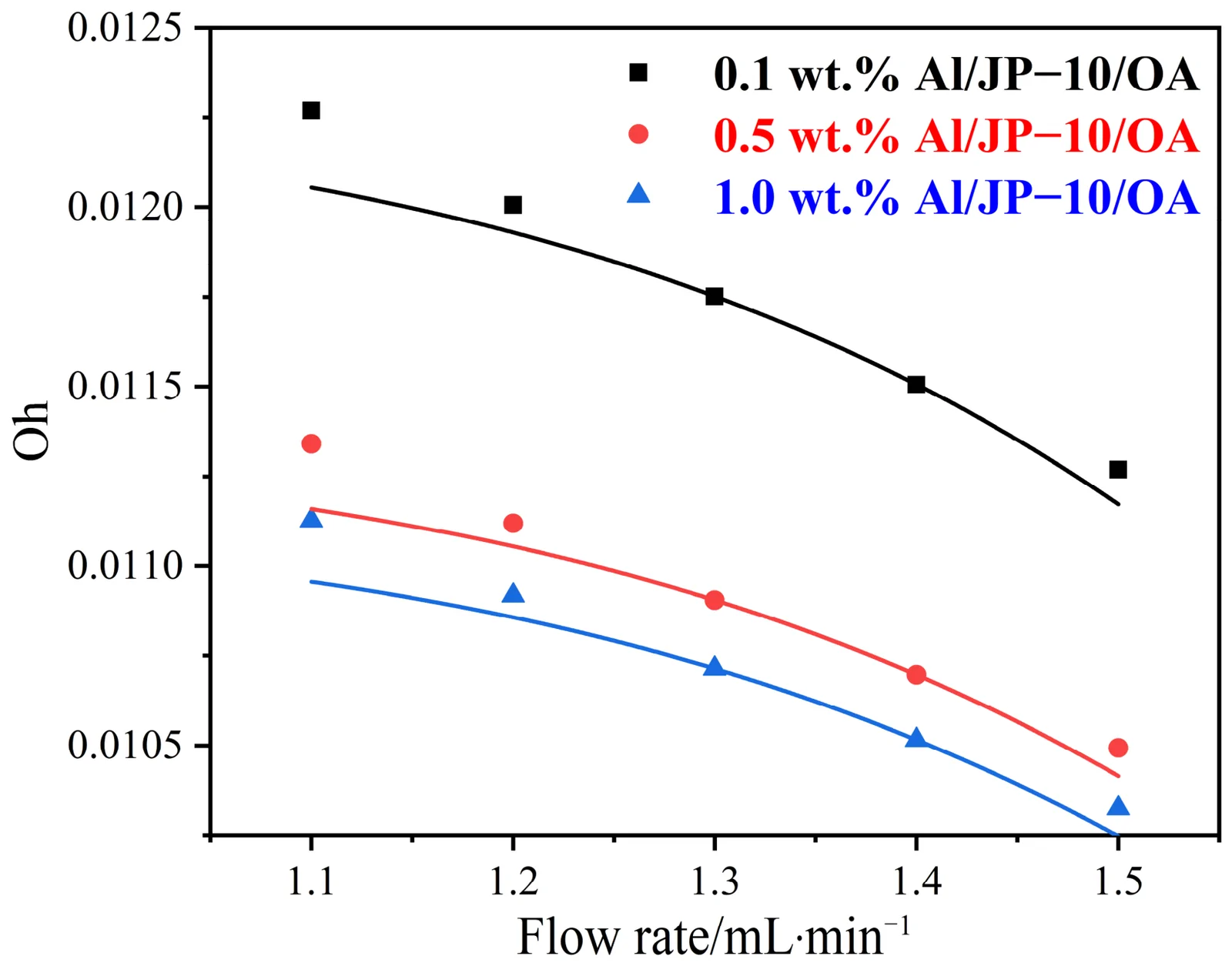
The *Oh* number of nanofluid droplets with different flow rates at 30 kHz.

The *Oh* values of 0.1 wt. %, 0.5 wt. % and 1.0 wt. % nanofluids at different flow rates at the frequency of 30 kHz are calculated as shown in Figure 12.

Figure 11 depicts the *Oh* number of nanofluids with different particle concentrations increases monotonically with the increase of driving frequency. Figure 12 shows that for all nanofluid concentrations, the *Oh* number decreases monotonically with increasing flow rate. The internal reason is that increasing flow rate will increase the droplet size generated in each single breakup event of the nanofluid. According to the *Oh* number definition and Formula (1), the relationship between *Oh* number and droplet flow rate and driving frequency can be obtained.

Combined with the physical properties of nanofluids and the change law of droplet size, this law covers the variations in droplet size with flow rate, driving frequency and nanoparticle concentration controlled by fluid viscous, inertial and surface tension effects, it can be seen that the *Oh* number decreases monotonically with the increase in particle concentration, which is completely consistent with the change trend of droplet size. It is confirmed from the quantitative level that the increase in viscous force is the core driving mechanism for the increase in droplet size of nanofluids.

The increase in the concentration of aluminum nanoparticles will increase the viscosity, density and surface tension of the fluid simultaneously. The increase in fluid viscosity will hinder the necking process of the liquid jet and prolong the jet breaking time. A single breakup will wrap more liquids and eventually form larger droplets. In addition, under the same working conditions, the droplet size generated by the high concentration fluid is larger and the *Oh* number is lower. Under the condition of a high *Oh* number, viscous dissipation is the main resistance of jet breakup. The surface tension and fluid inertia play a dominant role in the stability of the jet under low *Oh* number conditions. At the same time, the control linearity of the liquid flow rate on the *Oh* number is better than that of the piezoelectric driving frequency.

From the perspective of the Weber number (*We*), which represents the ratio of inertial force to surface tension and reflects the fragmentation tendency of the jet [37]:

(5) $$We(\varphi )=\frac{\rho_{nf}(\varphi )u_{nf}^{2}D}{\gamma_{nf}(\varphi )}$$

**Figure 13:**
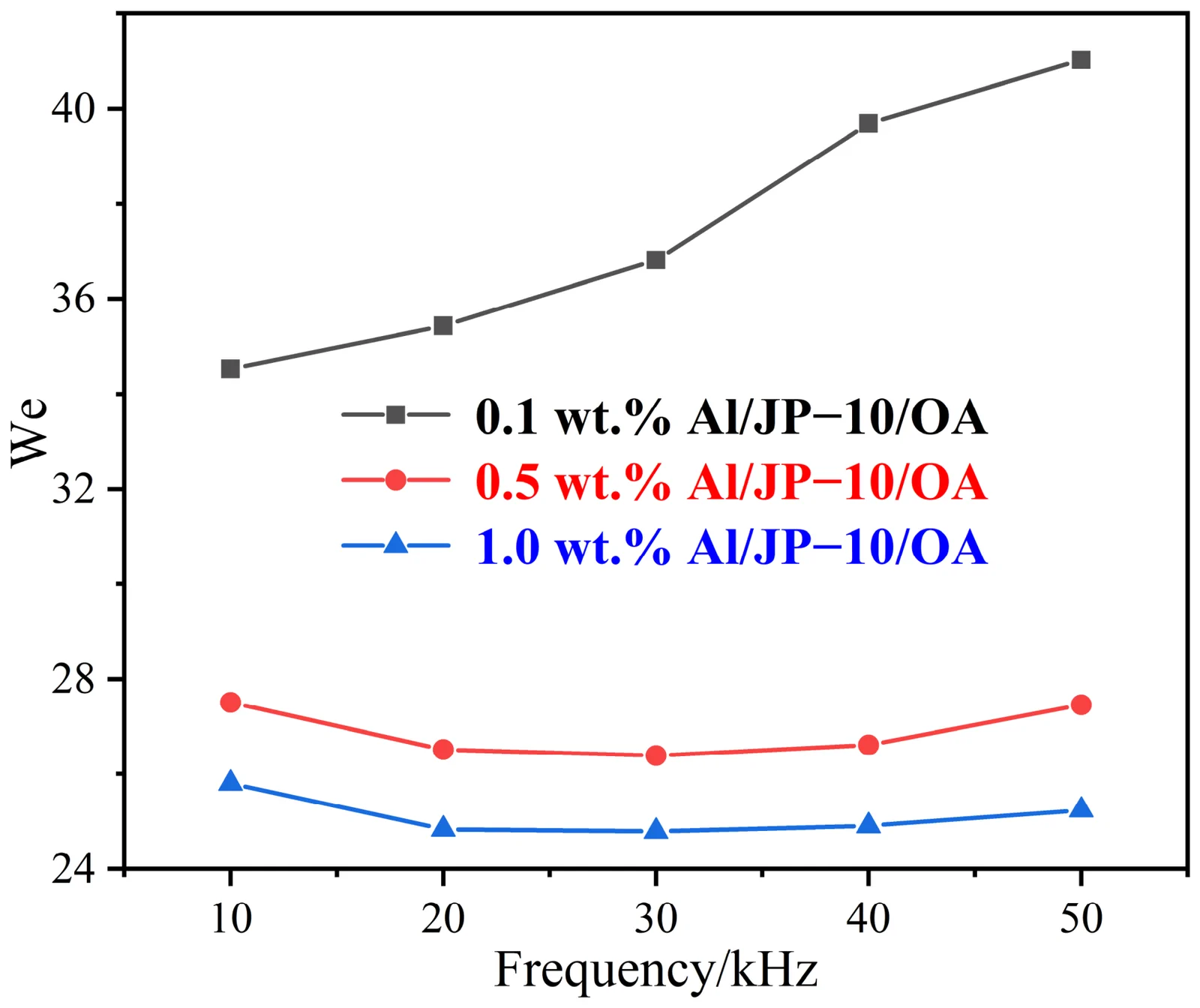
The *We* number of nanofluid droplets at different frequencies at 1.3 mL/min.

A smaller *We* number means stronger stabilization of surface tension, more difficult jet breakup, and larger droplet size. The *We* number of each fuel at different frequencies at the flow rate of 1.3 mL/min are calculated and shown in Figure 13.

**Figure 14:**
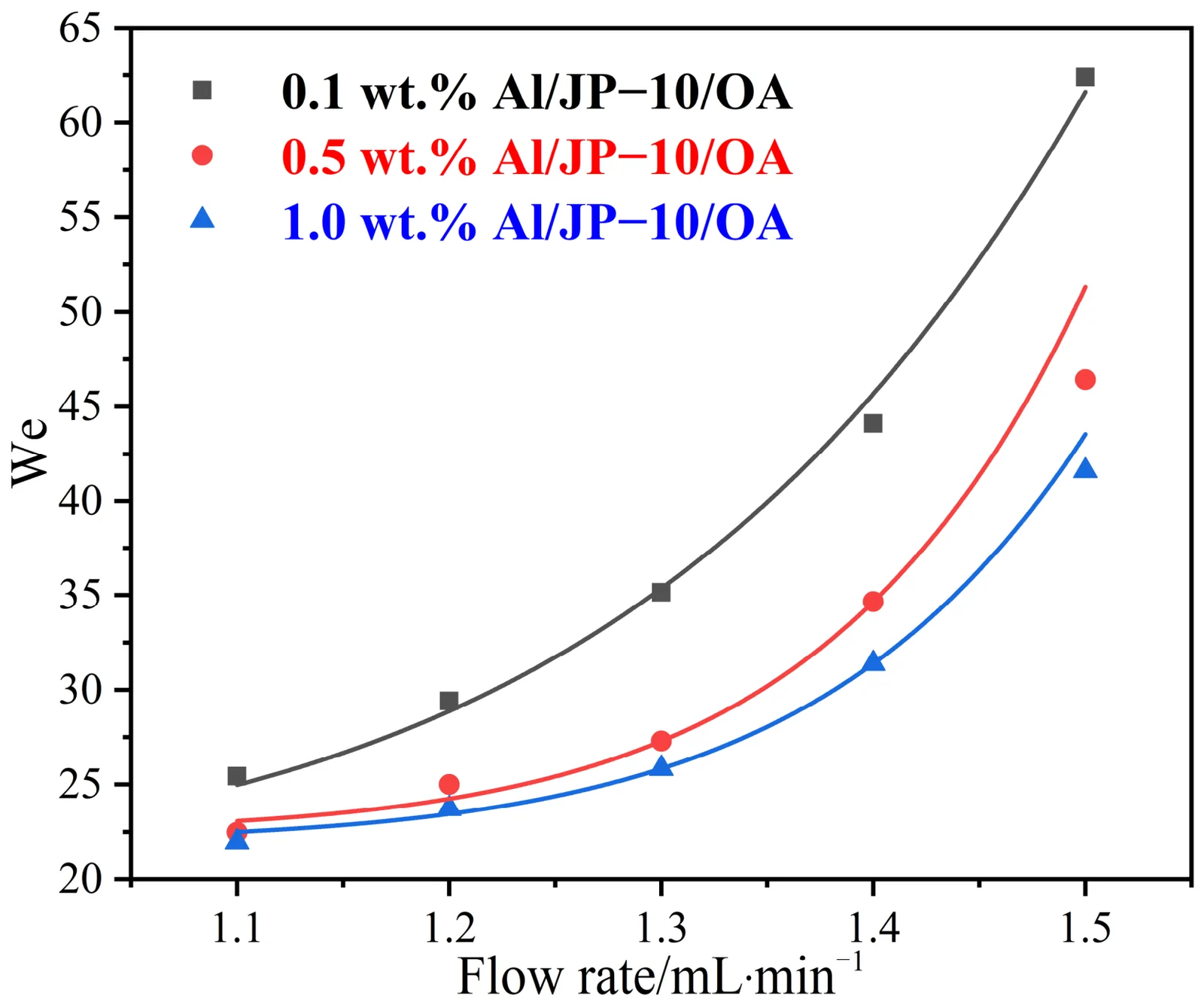
The *We* number of nanofluid droplets with different flow rates at 30 kHz.

The *We* values of 0.1 wt. %, 0.5 wt. % and 1.0 wt. % nanofluids at different flow rates at the frequency of 30 kHz are calculated as shown in Figure 14.

The We number exhibits a power-law growth with the rise in feed flow rate, obeying the scaling relation $We\propto q^{7/3}$, yet it exhibits a distinctive variation trend with driving frequency. For low-concentration nanofluids, the We number rises monotonically as driving frequency increases. However, as particle concentration grows, the *We* number of nanofluids first declines and then rebounds with the elevation of driving frequency. The increase in flow rate can greatly increase the jet injection velocity, and the square of velocity dominates the increase in *We*, and the linear control effect is stable. The increase in driving frequency synchronously reduces the droplet size and increases the flow rate. The surface tension of low concentration fluid is small, and the flow rate gain is dominant, which makes *We* continue to rise. The surface tension of high concentration fluid increases significantly. The reduction in low frequency particle size inhibits *We*, and the increase in high frequency flow rate promotes *We* to rise again. The increase in aluminum particle concentration increases the fluid density and surface tension synchronously. The weakening effect of surface tension on *We* is dominant, and the contribution of inertial force to jet breakup is weakened. The higher the concentration, the more difficult it is for the droplets to break up by inertia.

The lower *We* number of nanofluids indicates that the relative effect of surface tension is enhanced and the jet breakup difficulty is increased, which is another important reason for their larger droplet size. It is worth noting that the surface activity of oleic acid should theoretically reduce surface tension and increase the *We* number, but the inertial effect caused by increased particle density and the damping effect caused by increased viscosity dominate, finally resulting in decreased *We* number and enlarged droplet size.

#### 3.3.2. Rheological Modification Under High-Frequency Shear

Static viscosity characterizes fluid properties at low shear rates [38], while piezoelectric-driven droplet generation is a typical high-frequency high-shear process, with the shear rate at the nozzle reaching 10^4^–10^5^ s^−1^. Al/JP-10/OA nanofluids belong to pseudoplastic non-Newtonian fluids with significant shear-thinning characteristics. Under high shear rates, the agglomerate structures formed by particles are broken up by shear force, and the apparent viscosity of the fluid is significantly lower than the static measured value. The higher the particle concentration, the more significant the shear-thinning effect. This effect alleviates the hindrance of viscosity increase on jet breakup to a certain extent, which explains why the actual increase amplitude of droplet diameter in experiments is smaller than the theoretical prediction based on static viscosity. Meanwhile, the shear-thinning characteristic also affects the stability of droplet generation. Fluids with high viscosity at low shear rates experience rapid viscosity reduction in the high-shear nozzle region, making the jet breakup process more sensitive to fluctuations in flow rate and frequency. This is also an important internal reason for the rapid degradation of monodispersity of high-concentration nanofluids when the working condition deviates from the optimal range.

#### 3.3.3. Interfacial Adsorption Effect on Jet Stability

The behavior of the gas–liquid interface is a key factor determining jet stability [39]. Aluminum nanoparticles have high surface energy and will spontaneously migrate to and adsorb on the gas–liquid interface, forming a particle-enriched interfacial layer that modulates the jet breakup process. At low concentrations, particles are sparsely and uniformly distributed at the interface, and the mechanical strength improvement of the interfacial layer is limited, having little effect on jet stability. The jet breakup is still dominated by uniform Rayleigh instability, so the droplet uniformity is good. With the increase in concentration, more particles adsorb at the interface, forming a particle interfacial film with certain viscoelasticity. On the one hand, it increases the interfacial cohesion energy, raises the interfacial resistance of jet breakup, and promotes the enlargement of droplet size. On the other hand, the heterogeneity of the interfacial film induces local instability, causing random shifts in the breakup position and increasing the dispersion of droplet size distribution, as illustrated in Figure 4 and Figure 8. In addition, there is competitive adsorption between oleic acid molecules and aluminum particles at the interface. Oleic acid, as a surfactant, reduces surface tension and promotes jet breakup, while aluminum particles enhance interfacial strength and inhibit breakup [40]. For pure JP-10, the surface tension is 23.69 mN/m. For Al/JP-10/OA nanofluids, surface tension increases with Al mass fraction: 25.31 mN/m at 0.1 wt. % Al, 29.24 mN/m at 0.5 wt. % Al, and 30.33 mN/m at 1.0 wt. % Al. Benefiting from competitive adsorption between oleic acid molecules and aluminum nanoparticles at the gas–liquid interface, the growth rate of surface tension gradually decreases with increasing particle concentration. This phenomenon supports the above-mentioned interfacial mechanism and results in the nonlinear growth rate of droplet size, which can be verified from Figure 5 and Figure 9.

#### 3.3.4. Particle Agglomeration and Monodispersity Degradation

The degradation of size uniformity is directly related to the agglomeration behavior of nanoparticles [41]. At low concentration (0.1 wt. %), the average distance between particles is large, and the dispersion effect of Brownian motion is stronger than the van der Waals attraction. Particles are uniformly dispersed, and the fluid approximates a homogeneous system. At this time, jet breakup is dominated by uniform Rayleigh instability with highly consistent breakup periods, so the size distribution is narrow and the monodispersity is excellent, with a *Span* value of only 0.12. When the concentration rises above 0.5 wt. %, the inter-particle distance decreases significantly, and the attractive van der Waals force gradually dominates, leading to the formation of micro-agglomerates with varying sizes. These agglomerates damage droplet uniformity through two pathways. (1) Local physical property disturbance: the local viscosity and density near agglomerates are significantly higher than those of the bulk fluid, which inhibits the growth of local disturbances, delays jet breakup, and forms oversized droplets. (2) Interfacial instability induction: when agglomerates migrate to the gas–liquid interface with the liquid flow, they cause local morphological distortion of the interface and induce premature local instability and breakup, forming tiny satellite droplets [42].

The combined effect of the two significantly enhances the randomness of jet breakup, widens the size distribution and degrades monodispersity. When the concentration reaches 1.0 wt. %, the number and size of agglomerates further increase, which may also cause local adhesion and slight blockage on the inner wall of the nozzle, aggravating the fluctuation of ejection flow and leading to a simultaneous increase in velocity dispersion. This phenomenon is consistent with the droplet size distribution diagrams (Figure 4 and Figure 8) and droplet morphology images (Figure 3 and Figure 7) presented in this work.

#### 3.3.5. Energy Dissipation Mechanism of Droplet Ejection

Droplet ejection velocity is determined by the balance between the energy transfer efficiency of piezoelectric vibration and flow damping. Aluminum particle doping reduces the energy transfer efficiency from three aspects, leading to a monotonous decrease in ejection velocity with increasing concentration [43]. (1) Enhanced viscous dissipation: The increase in fluid viscosity enlarges the frictional resistance and local resistance in the liquid chamber and nozzle channel. More vibration energy is dissipated as heat through viscosity, and the proportion converted into macroscopic kinetic energy of droplets decreases. (2) Particle damping effect: There exists relative motion and momentum exchange between nanoparticles and the base liquid. Vibration energy is dissipated through particle–fluid interaction, and the dissipation effect becomes more significant with higher concentration. (3) Increased inertial impedance: The elevated fluid density increases the inertia per unit volume of liquid, resulting in a lower velocity increment under the same pressure impulse. This trend is consistent with the experimental results presented in Figure 6 and Figure 10.

## 4. Conclusions

This study established a piezoelectric drop-on-demand system integrated with phase Doppler anemometry and high-speed imaging to investigate Al/JP-10/OA nanofluids (0.1–1.0 wt. % Al). The main conclusions are:

(1) Droplet size decreases with driving frequency and increases linearly with flow rate, while ejection velocity rises with both parameters but saturates at high frequencies due to pressure recovery limits. Optimal monodispersity is achieved at 30 kHz and 1.1–1.5 mL/min for concentrations ≤0.5 wt. %.

(2) Increasing particle concentration (0.1–1.0 wt. %) raises fluid density/viscosity, leading to a 5.36% larger droplet diameter and 12.4% lower ejection velocity (at 1.3 mL/min, 30 kHz). Low-concentration (0.1 wt. %) nanofluids maintain excellent uniformity (*Span* < 0.13), but higher concentrations cause agglomeration-induced broadening and degraded monodispersity.

(3) Concentration modulates breakup via coupled mechanisms: macroscopically, higher viscosity raises the Ohnesorge number and lowers the Weber number, hindering jet breakup and increasing droplet size; microscopically, shear-thinning partially offsets viscosity rise, while particle adsorption/agglomeration destabilize the jet. Energy dissipation (viscous, damping, inertial) intensifies with loading, attenuating ejection velocity.

These results provide quantitative guidance for nanofluid atomization design and single-droplet combustion experiments. Future work will extend to evaporation/combustion under high-temperature/pressure conditions.

## Figures and Tables

**Table 1:** Physical properties of pure JP-10 and Al/JP-10/OA nanofluids at 25 °C.

Fuel Type	Density (kg/m^3^)	Dynamic Viscosity (mPa·s)	Surface Tension (mN/m)
JP-10	940	0.918	23.69
0.1 wt. % Al/JP-10/OA	940	0.920	25.31
0.5 wt. % Al/JP-10/OA	942	0.929	29.24
1.0 wt. % Al/JP-10/OA	945	0.945	30.33

## Data Availability

The original contributions presented in this study are included in the article. Further inquiries can be directed to the corresponding author.
